# The life satisfaction of intern nursing students and its relationship with flourishing: a latent profile analysis

**DOI:** 10.3389/fpsyg.2026.1887731

**Published:** 2026-09-23

**Authors:** Ziyi Liao, Jin Zou, Fang Yu

**Affiliations:** Department of Cardiology, The First Affiliated Hospital, Hengyang Medical School, University of South China, Hengyang, Hunan, China

**Keywords:** clinical internship, flourishing, intern nursing students, latent profile analysis, life satisfaction

## Abstract

**Background:**

Clinical internship represents a demanding transition period for nursing students, during which maintaining positive psychological wellbeing is important for both personal development and professional preparation. Although life satisfaction has been increasingly recognized as an important indicator of wellbeing among nursing students, existing research has largely treated it as a homogeneous construct, with limited attention to potential differences in life satisfaction patterns within this population. Moreover, little is known about how distinct life satisfaction profiles are associated with flourishing, a broader indicator of positive psychological functioning.

**Aims:**

This study aims to identify and describe the potential categories of life satisfaction among intern nursing students and to elucidate the flourishing levels associated with each category.

**Methods:**

A cross-sectional study was conducted using three data collection instruments: a self-designed general information questionnaire, the Chinese version of the Flourishing Scale, and the Satisfaction with Life Scale. Latent profile analysis, descriptive analysis, and Welch's test were performed on data collected from 315 intern nursing students from a tertiary hospital in Hunan Province, China.

**Results:**

The results of the latent profile analysis (LPA) indicated that a three-profile model was the most appropriate, supporting the existence of three distinct life satisfaction profiles among intern nursing students: the low life satisfaction group (8.2%), the moderate life satisfaction group (58.4%), and the high life satisfaction group (33.4%), with a relatively high entropy value (0.952). The results demonstrated a good profile solution, with significant differences among the three profiles. Welch's test further revealed significant differences in flourishing levels among the different life satisfaction groups, with the high life satisfaction group scoring higher than the moderate group, and the moderate group scoring higher than the low life satisfaction group.

**Conclusion:**

Our results revealed the heterogeneity of life satisfaction among intern nursing students, indicating that those in the high life satisfaction group tended to exhibit higher levels of flourishing. These findings provide important implications and reference value for nursing educators, who can develop customized educational programs based on the different types of life satisfaction and the corresponding levels of flourishing among nursing students.

## Introduction

In recent years, the mental health and wellbeing of nursing students have received increasing attention. Nursing students, particularly those undergoing clinical internships, face multiple challenges, including demanding academic and clinical responsibilities, complex patient-care situations, and emotional stress associated with clinical practice (Liu et al., 2022; Alzayyat and Al-Gamal, 2014). These challenges may adversely affect their psychological wellbeing and contribute to burnout and lower satisfaction with life (Priano et al., 2018). Life satisfaction is an important component of subjective wellbeing and reflects an individual's overall cognitive evaluation of the quality of their life (Wright and Cropanzano, 2000). According to Diener's conceptualization of subjective wellbeing, subjective wellbeing comprises both cognitive and affective evaluations of life, including life satisfaction, positive affect, and negative affect (Diener et al., 1999). Within this framework, life satisfaction represents the cognitive component of subjective wellbeing and reflects how individuals judge their lives according to their own standards and expectations. Therefore, understanding life satisfaction among nursing students may provide important insight into their overall psychological adjustment during the transition from academic learning to clinical practice (Williams et al., 2018).

From the perspective of positive psychology, mental health is not defined solely by the absence of psychological distress but also by the presence of positive functioning and optimal wellbeing (Keyes, 2002). Building on this perspective, Seligman proposed the WellBeing Theory, in which flourishing represents a broader state of optimal human functioning rather than a single feeling of happiness or life satisfaction (Seligman, 2011). The theory operationalizes wellbeing through the PERMA model, comprising five core elements: Positive Emotion, Engagement, Relationships, Meaning, and Accomplishment (Butler and Kern, 2016). These elements are conceptually distinct but collectively contribute to individuals' capacity to function well and lead meaningful and fulfilling lives. Whereas, Diener's subjective wellbeing framework emphasizes individuals' cognitive and affective evaluations of their lives, the PERMA framework extends the understanding of wellbeing to include broader dimensions of psychological and social functioning (Seligman, 2018). Accordingly, flourishing can be viewed as a more comprehensive indicator of positive mental health, encompassing but extending beyond satisfaction with life (Huppert and So, 2013). Previous studies have associated flourishing among university students with greater academic engagement, resilience, and social connectedness, as well as lower levels of psychological distress (Antaramian, 2015; Van Zyl and Stander, 2019). For nursing students, flourishing may therefore represent an important indicator of positive adaptation during clinical training (Yeşi Ltepe et al., 2022; Darch et al., 2019).

However, nursing students are unlikely to constitute a psychologically homogeneous population. Previous studies have reported substantial variation in life satisfaction among nursing students, with some students experiencing relatively favorable levels of wellbeing while others report considerably lower life satisfaction (Tsusaki et al., 2024). Nevertheless, most previous studies have examined life satisfaction using a variable-centered approach, focusing primarily on overall scores, associated factors, or relationships between variables (Temiz et al., 2026). Such approaches may obscure meaningful heterogeneity in how different dimensions or indicators of life satisfaction co-occur within individuals. Identifying distinct patterns of life satisfaction may therefore provide a more nuanced understanding of nursing students' subjective wellbeing and help distinguish subgroups with different psychological characteristics.

Latent Profile Analysis (LPA) is a person-centered statistical approach that identifies relatively homogeneous subgroups of individuals according to their response patterns across a set of observed indicators (Hong et al., 2023). Compared with conventional variable-centered analyses, LPA can reveal unobserved heterogeneity within a population and characterize distinct configurations of life satisfaction. Examining whether these profiles differ in flourishing may further clarify how different patterns of subjective life evaluation are associated with broader positive psychological functioning. Therefore, this study aimed to identify latent profiles of life satisfaction among intern nursing students and examine differences in flourishing across these profiles. By integrating the subjective wellbeing perspective with the PERMA-based conceptualization of flourishing, this study provides a person-centered understanding of wellbeing among nursing students and may inform more targeted strategies to support their psychological wellbeing during clinical internship.

## Materials and methods

This was a cross-sectional survey. Intern nursing students from a tertiary hospital in Hengyang City, China, were selected as the study participants. Inclusion criteria: clinical internship duration of ≥2 months; informed consent to participate in the survey. Exclusion criteria: students who were on sick leave, personal leave, or otherwise absent from the hospital during the investigation period.

### Survey tools

#### General information survey questionnaire

The general information questionnaire collected data on gender, only-child status, educational level, place of residence, voluntary choice of nursing as a major, willingness to engage in nursing work after graduation, intention to pursue further education, place of residence during the clinical internship, paternal educational attainment, and maternal educational attainment.

#### Life satisfaction scale

The Life Satisfaction Scale was developed by Diener et al. (1985). It was introduced to China by Hua-Jian (2008). This scale consists of 5 items and is scored on a 7-level scale ranging from 1 (very inconsistent) to 7 (very consistent). The higher the score, the higher the level of life satisfaction. The Cronbach's α coefficient of the scale is 0.81 (Hua-Jian, 2008).

#### The flourishing scale

Compiled by Diener et al. (2010), it is used to assess an individual's overall wellbeing. The Flourishing Scale is a single-dimensional scale consisting of 8 items, using a Likert 7-point scale (ranging from 1 to 7 points for strongly disagree to strongly agree), with a total score ranging from 8 to 56 points. The higher the score, the higher the level of mental health. The Cronbach a coefficient measured among college students is 0.870 (Diener et al., 2010).

#### Survey methods

Data were collected from July to August 2025 using Wenjuanxing. The questionnaire measurement will take approximately 10 min to complete. After obtaining the consent of the director of the nursing department, the questionnaire link was forwarded through the head nurses of each department. Intern nurses are willing to participate. Click the link to fill in. All options are set to anonymous.

#### Sample size

The sample size was determined a priori according to both the number of questionnaire items and the requirements of latent profile analysis. Based on a recommended participant-to-item ratio of 5–10:1, the 13 scale items required 65–130 participants (Veronese et al., 2025). Given the greater sample size requirement of latent profile analysis, the minimum effective sample size was set at 300 participants (Nylund-Gibson and Choi, 2018). Of the 328 questionnaires collected, 315 were valid and included in the final analysis, exceeding the prespecified minimum sample size.

#### Statistical analysis

The data were analyzed using SPSS 27.0 and Mplus 8.3 software. Firstly, use LPA to determine the potential categories of life satisfaction of trainee nurses; Commonly used adaptive fitting indicators include Akaiike Information Criterion (AIC), Bayesian Information Criterion (BIC), and Sample Corrected BIC (aBIC). The smaller the values of these three indicators are, the better the fit between the data and the model will be. The range of entropy value is 0 to 1. The larger the entropy value, the higher the classification accuracy. When the *P*-values corresponding to the Bootstrap likelihood ratio test (BLRT) based on the Bootstrap method and the Lo Mendell Rubin likelihood ratio test (LMR) are significant (*P* < 0.05), it indicates that the performance of the *K*-class model is superior to that of the *k*-1 class model. Secondly, SPSS 27.0 was used for statistical analysis of the data. The Kolmogorov-Smirnov (KS) test was used to examine the normality of the data distribution (*P* < 0.05). Counting data are expressed in terms of frequency and percentage, and represent the median and interquartile intervals of the measurement data. The Welch's test was used for intergroup comparative analysis. The difference was statistically significant when *P* < 0.05.

## Results

### General information about intern nursing students

A total of 315 intern nursing students were included in the study. Most participants were female (89.5%), non-only children (88.9%), and had voluntarily chosen nursing as their major (72.1%). Nearly half had a junior college education (48.6%) and resided in rural areas (48.6%). More than half expressed willingness to engage in nursing work after graduation (57.8%), and 63.5% intended to pursue further education. During the internship, 67.9% lived in rental accommodation. With regard to parental educational attainment, junior high school was the most common educational level among both fathers (46.3%) and mothers (47.0%). Detailed characteristics are presented in Table 1.

**Table 1:** General information of intern nursing students (*n* = 315).

Variable	Classification	Number of cases (composition ratio)
Gender	Male	33(10.5)
	Female	282(89.5)
Only child	Yes	35(11.1)
	No	280(88.9)
Education	Secondary vocational school	41(13.0)
	Junior college	153(48.6)
	Undergraduate	98(31.1)
	Graduate student	23(7.3)
Place of residence	Rural areas	153(48.6)
	County town	89(28.3)
	City	73(23.2)
Voluntarily choose the nursing major	Yes	227(72.1)
	No	88(27.9)
The willingness to engage in nursing work after graduation	Unwilling	20(6.3)
	Not clear	113(35.9)
	Willing	182(57.8)
Intention for further education	Yes	200(63.5)
	No	115(36.5)
Place of residence during internship	Rental house	214(67.9)
	Relatives‘ and friends' homes	10(3.2)
	Dormitory	32(10.2)
	Own home	59(18.7)
The educational attainment of the father	Primary school and below	38(12.1)
	Junior high school	146(46.3)
	High school or technical secondary school	86(27.3)
	Junior college	32(10.2)
	Undergraduate	13(4.1)
The educational attainment of the monther	Primary school and below	63(20.0)
	Junior high school	148(47.0)
	High school or technical secondary school	76(24.1)
	Junior college	21(6.7)
	Undergraduate	7(2.2)

### Description of life satisfaction and heart satisfaction scores

The specific situation is shown in Table 2.

**Table 2:** Scores of life satisfaction and flourishing among intern nursing students (*n* = 315).

Variable	Minimum value	Maximum value	[M(P25, P75)]
My life roughly conforms to my ideals	1	7	4[4,6]
My living conditions are very good	1	7	5[4,6]
I'm satisfied with my life	1	7	5[4,6]
So far, I have got the important things I want in life	1	7	4[4,5]
If life could start over, I wouldn't want to change anything either	1	7	4[3,5]
Total score of life satisfaction	5	35	21[19,27]
Total score of flourishing	8	56	39[33,46]

M, median; P25, 25th percentile; P75, 75th percentile.

### Potential profile analysis of life satisfaction of intern nursing students

One to five potential category models were developed based on the five items of life satisfaction. Table 3 shows the results of the fitting indicators of the LPA model. From Model 1 to Model 5, AIC, BIC and aBIC gradually decrease, while entropy gradually increases. However, the LMR of models 4 and 5 is greater than 0.05, indicating that there is no classification significance. Therefore, the life satisfaction of intern nursing students is ultimately classified into three categories.

**Table 3:** Potential profile fitting index of life satisfaction of intern nursing students (*n* = 315).

Model	LL	AIC	BIC	aBIC	LMR	BLRT	Entropy	Category probability
1	−2764.376	5548.751	5586.277	5554.560	–	–	–	1
2	−2369.208	4770.417	4830.458	4779.710	0.0003	0.0000	0.902	0.61270/0.38730
3	−2198.435	4440.871	4523.428	4453.650	0.0084	0.0000	0.952	0.08254/0.33333/0.58413
4	−2107.428	4270.856	4375.928	4287.120	0.5608	0.0000	0.918	0.24762/0.06032/0.44444/0.24762
5	−2016.853	4101.707	4229.294	4121.455	0.0585	0.0000	0.993	0.04127/0.41905/0.20635/0.12063/0.21270

### Characteristics, distribution and naming of potential categories of life satisfaction for intern nursing students

The average values of the three categories on the five items of the life satisfaction questionnaire for intern nursing students are shown in Figure 1. Classification results based on latent categories. The average differences among the three potential categories of trainee nurses in the five items were statistically significant, and the overall morphological trends of the two subtypes C2 and C3 were consistent. In the potential category group, the scores of all dimensions in category C1 were lower than those in other categories, accounting for 8.2%. Therefore, it is called the “Low Life Satisfaction Group”. Category C2 scores higher than C1 and C3 in all dimensions, accounting for 33.4%. Therefore, it is called the “High Life Satisfaction Group”. Category C3 scores between C1 and C2 in all dimensions, accounting for 58.4%. Therefore, it is called the “Middle Life Satisfaction Group”.

**Figure 1:**
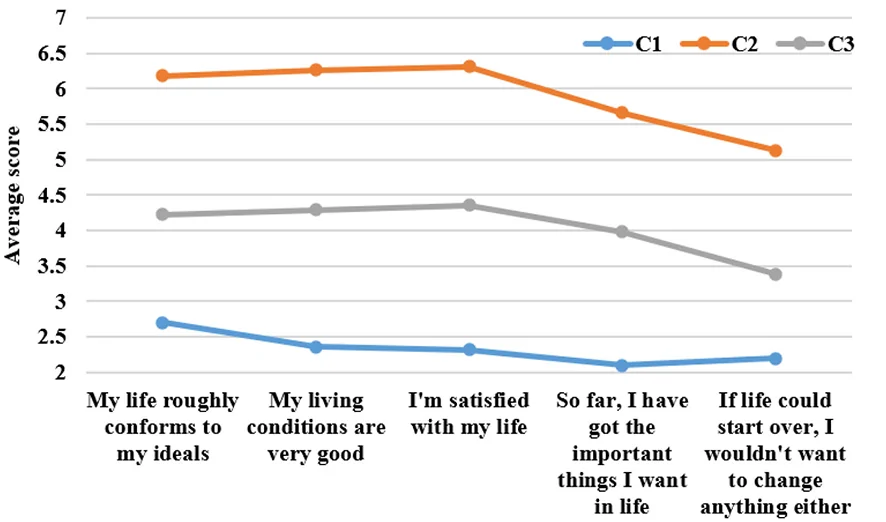
Distribution of three potential characteristics of life satisfaction of trainee nurses (C1 8.2%, C2 33.4%, C3 58.4%).

### Comparison of life satisfaction and flourishing across latent profiles

Differences in the five life satisfaction items, total life satisfaction score, and total flourishing score were compared across the three latent profiles. As shown in Table 4, statistically significant differences were observed across the three profiles for all five life satisfaction items, total life satisfaction score, and total flourishing score (all *P* < 0.001).

**Table 4:** Comparison of life satisfaction and flourishing across latent profiles among intern nursing students (*n* = 315).

Items	C1	C2	C3	H	*P*
My life roughly conforms to my ideals	3[2,4]	6[6,7]	4[4,5]	326.778	< 0.001
My living conditions are very good	3[1,3]	6[6,7]	4[4,5]	462.051	< 0.001
I'm satisfied with my life	2[1,3]	6[6,7]	4[4,5]	462.778	< 0.001
So far, I have got the important things I want in life	2[1,3]	6[5,6]	4[4,4]	146.166	< 0.001
If life could start over, I wouldn't want to change anything either	1[1,3]	6[4.6]	4[3,4]	65.860	< 0.001
Total score of life satisfaction	13[9,14]	30[27,31]	20[19,22]	454.370	< 0.001
Total score of flourishing	29.5[23,33.5]	48[44.5,52]	35[32,40]	181.846	< 0.001

## Discussion

This study applied latent profile analysis (LPA) to identify the potential subgroups of life satisfaction among intern nursing students. Three distinct categories were identified: the low life satisfaction group (8.2%), the moderate life satisfaction group (58.4%), and the high life satisfaction group (33.4%). The total scores and dimensional scores of life satisfaction differed significantly across the three profiles, suggesting the existence of heterogeneity in life satisfaction among nursing students. Moreover, the flourishing scores were significantly different among these potential categories, with the high life satisfaction group reporting the highest flourishing levels, followed by the moderate and low life satisfaction groups. This result partially supports the validity of the three-profile classification and demonstrates that higher life satisfaction is associated with greater psychological flourishing.

Overall, the level of life satisfaction of the trainee nurses in this study was at a moderately low level, lower than the previous research results (Turan et al., 2020; Kim, 2019). This result may be closely related to the special learning and working circumstances of the intern nursing students. Firstly, the internship stage is a crucial period for students to transition from the campus to the clinical environment. They are confronted with multiple challenges such as the transformation of identity and role, the increase of clinical work pressure, and the complication of the doctor-nurse relationship (Zhang et al., 2025; Deng et al., 2022). In such an environment, intern nursing students are prone to experiencing adaptation difficulties, fatigue or career confusion, thereby reducing their life satisfaction. Secondly, some students lack a sense of identification with the nursing major. In this study, 27.9% of the nursing students did not voluntarily choose the nursing major, which may further affect their life experience and satisfaction level (Zhouchen et al., 2024).

From the perspective of latent category characteristics, students in the high life satisfaction group generally scored higher in the dimensions of “living conditions,” “overall satisfaction,” and “ideal realization.” They are more likely to experience a sense of achievement and fulfillment in both study and daily life, demonstrating greater psychological resources and self-efficacy. These positive psychological traits contribute to enhancing their level of flourishing. In contrast, students in the low life satisfaction group often experience a gap between their life ideals and reality, lack a sense of control over their lives and sufficient social support, which leads to significantly lower flourishing scores. This finding aligns with the perspective of positive psychology, which posits that an individual's level of subjective wellbeing is closely related to their positive psychological functioning (Fredrickson and Losada, 2005). In addition, according to Diener's theory of happiness, life satisfaction represents an individual's cognitive assessment of the overall quality of life, while flourishing reflects the optimal state of an individual's psychological function. The two promote each other and jointly constitute an important component of positive mental health (Diener et al., 1999; Keyes, 2002).

### Research significance and clinical guidance

This study employed latent profile analysis to identify three distinct potential categories of life satisfaction among intern nursing students—namely, the Low Life Satisfaction Group, Moderate Life Satisfaction Group, and High Life Satisfaction Group. The results revealed significant differences in flourishing levels across these categories, with the overall life satisfaction level being moderately low. Importantly, this study is the first to uncover the heterogeneity of life satisfaction among intern nursing students, moving beyond traditional variable-centered approaches. Adopting a person-centered analytical perspective, it provides a more comprehensive understanding of the stratified characteristics of psychological wellbeing among nursing interns. Furthermore, by examining the differences in flourishing levels across life satisfaction profiles, this study offers new insights into the positive psychological mechanisms that influence nursing students' adaptation and growth during clinical internships.

In terms of educational and managerial practice, the findings have three main implications. First, nursing educators and administrators should place greater emphasis on the life satisfaction of intern nursing students and their close association with psychological flourishing. Efforts should be made to optimize the internship environment, foster supportive teacher-student relationships, and provide emotional and career guidance to enhance students' sense of control and professional identity, thereby promoting their positive psychological development. Second, differentiated educational strategies should be developed for students in different life satisfaction categories. For instance, those in the low life satisfaction group may benefit from strengthened psychological care and stress management training to help them build realistic career expectations and life goals, whereas those in the high life satisfaction group should be offered further opportunities for self-realization and professional development to consolidate their positive psychological traits. Finally, nursing administrators are encouraged to integrate positive psychology principles into clinical education systems, creating an educational model that fosters a virtuous cycle of life satisfaction-flourishing-professional growth.

Overall, this study provides empirical evidence and theoretical insights for nursing education, highlighting the importance of simultaneously addressing life satisfaction and flourishing to promote internship adaptation, professional identity formation, and long-term commitment to the nursing profession.

### Limitations

This study has certain limitations. Firstly, this study adopted a cross-sectional design, which failed to reveal the dynamic characteristics of different types of life satisfaction over time. In the future, the stability of the results can be further verified through longitudinal studies. Secondly, the research subjects were only from a tertiary grade A hospital in Hengyang City, China, and the representativeness of the sample was limited. The extrapolation of the research conclusions needs to be further verified. In addition, the scales adopted in the research were all self-assessment tools, which might be affected by subjective biases, thereby limiting the objectivity of the research results to a certain extent. Future qualitative studies are also recommended to explore the underlying experiences, perceptions, and contextual factors that may contribute to differences in life satisfaction and flourishing among intern nursing students.

### Conclusion

This study employed latent profile analysis and identified three potential categories of nursing interns' life satisfaction: the “low life satisfaction group,” the “moderate life satisfaction group,” and the “high life satisfaction group.” The findings revealed distinct differences in psychological characteristics and flourishing levels among these groups. The results provide a new intervention perspective for nursing education and student management, emphasizing the importance of addressing the psychological characteristics of nursing interns with different levels of life satisfaction and implementing targeted psychological support and empowerment strategies to enhance their overall wellbeing and mental health.

## Data Availability

The raw data supporting the conclusions of this article will be made available by the authors, without undue reservation.
